# Daily low dose aspirin halves incident type 2 diabetes in elderly subjects with prediabetes: a five-year longitudinal cohort study in a real-word population

**DOI:** 10.1186/s12933-025-02802-9

**Published:** 2025-06-18

**Authors:** Maria Lembo, Valentina Trimarco, Daniela Pacella, Raffaele Izzo, Maria Virginia Manzi, Stanislovas S. Jankauskas, Roberto Piccinocchi, Paola Gallo, Carmine Morisco, Luca Bardi, Gaetano Piccinocchi, Stefano Cristiano, Giuseppe Giugliano, Giovanni Esposito, Gaetano Santulli, Bruno Trimarco

**Affiliations:** 1 Department of Advanced Biomedical Sciences, “Federico II” University, Via Pansini 5, Naples, Italy; 2 Department of Neuroscience, Reproductive Sciences, and Dentistry, “Federico II” University, Naples, Italy; 3 Department of Public Health, “Federico II” University, Naples, Italy; 4 Department of Medicine, Wilf Family Cardiovascular Research Institute, Einstein-Mount Sinai Diabetes Research Center (ES-DRC), Fleischer Institute for Diabetes and Metabolism (FIDAM), Albert Einstein College of Medicine, New York City, NY USA; 5 “Luigi Vanvitelli” Hospital, Naples, Italy; 6 International Translational Research and Medical Education (ITME) Consortium, Academic Research Unit, Naples, Italy; 7 Italian Society for Cardiovascular Prevention (SIPREC), Rome, Italy; 8 COMEGEN Primary Care Physician Cooperative, Italian Society of General Medicine (SIMG), Naples, Italy; 9 Department of Molecular Pharmacology, Einstein Institute for Aging Research, Institute for Neuroimmunology and Inflammation (INI), Albert Einstein College of Medicine, New York City, NY USA

**Keywords:** Aging, Aspirin, Diabetes mellitus, PSM, Prediabetes

## Abstract

**Background:**

Prediabetes represents the final stage on the glycemic spectrum before the onset of type 2 diabetes mellitus (T2DM), and delaying its progression offers a unique opportunity to address the growing T2DM epidemic.

**Methods:**

In this longitudinal cohort study, we investigated the effect of daily low-dose aspirin on the development of T2DM in individuals with prediabetes residing in Naples, Italy, who were followed by their primary care physicians between 2018 and 2022. Outcomes in the aspirin-treated group were compared with those in a control group not receiving aspirin, using data from the same database. Propensity score matching was employed to ensure comparability of covariates at baseline.

**Results:**

The primary outcome was the onset of T2DM, defined as a new diagnosis accompanied by antidiabetic prescriptions lasting more than 30 days. Gastrointestinal bleeding was assessed as the safety endpoint. Over the follow-up period, 488 new cases of T2DM were documented (15.6% of the total population), with 174 cases occurring in the aspirin group (22.3 per 1000 person-years) and 314 in the non-aspirin group (40.2 per 1000 person-years), indicating a significantly lower incidence of diabetes among aspirin-treated individuals. Given the difference in comorbidity rates between groups, a Cox regression analysis was conducted across the entire follow-up period, showing that aspirin use was associated with a 47% reduction in the risk of developing T2DM (HR 0.53, 95% CI 0.44–0.64, *p* < 0.001). However, aspirin use was also linked to an increased risk of gastrointestinal bleeding (4.9% vs 3.1%, *p* < 0.05). Kaplan–Meier survival curves confirmed a significantly lower cumulative incidence of T2DM in the aspirin-treated group (log-rank test *p* < 0.0001).

**Conclusions:**

Daily treatment with 100 mg aspirin was associated with approximately a 50% reduction in the incidence of new-onset T2DM, but also with an increased risk of gastrointestinal bleeding, in elderly individuals with prediabetes.

**Graphical abstract:**

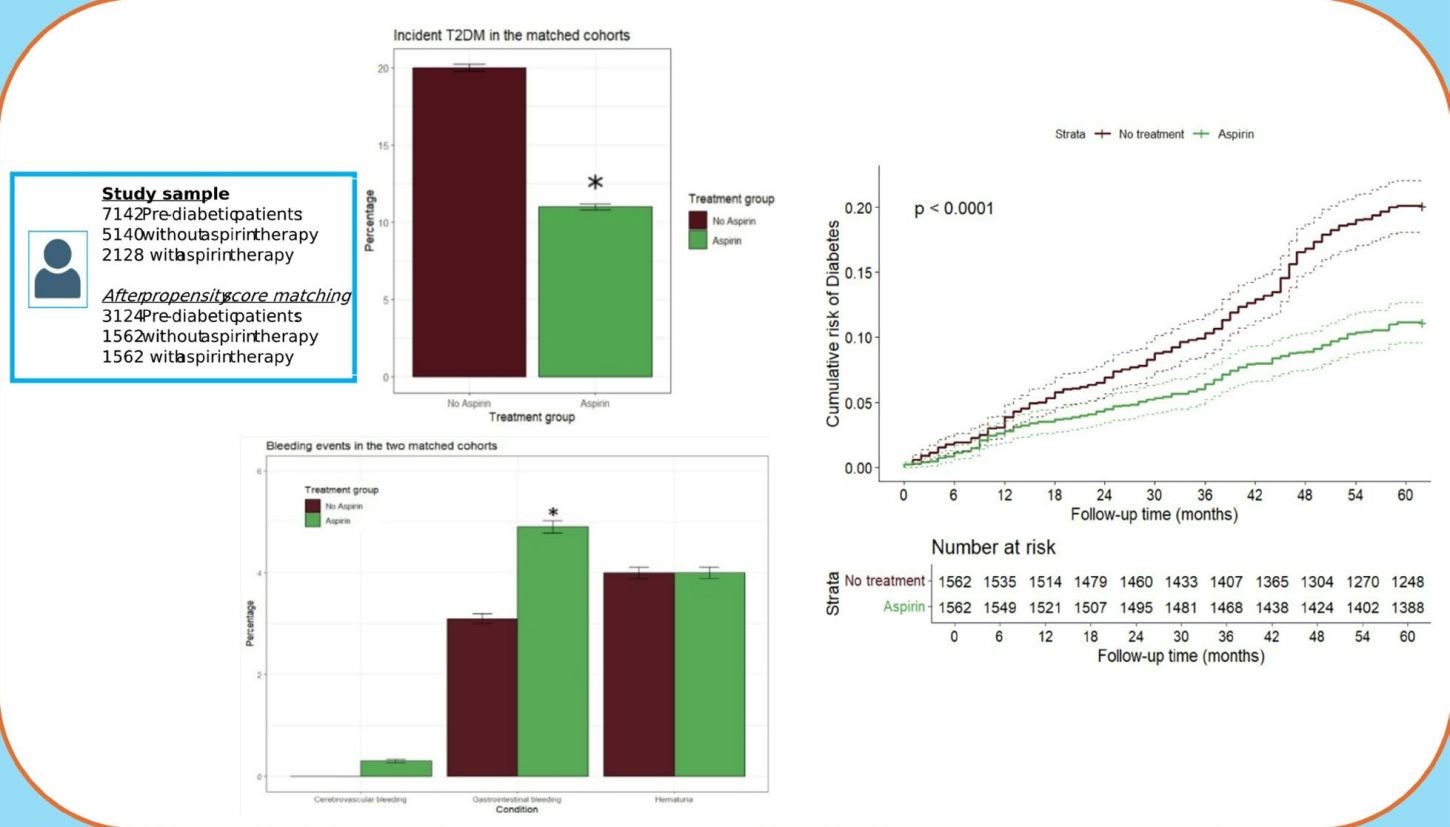

## Research Insights

**What is currently known about this topic?**

- Daily low-dose aspirin can slow increase in fasting plasma glucose.

**What is the key research question?**

- What is the effect of daily low-dose aspirin on incident diabetes in individuals with prediabetes?

**What is new?**

- Treatment with daily dose of 100 mg aspirin was associated with a reduction of about 50% of the incidence of new diabetes despite an increased risk of gastrointestinal bleeding in elderly subjects with prediabetes with a long-term follow-up.

**How might this study influence clinical practice?**

- Daily low-dose aspirin may help in reducing the burden of diabetes impact in prediabetic individuals.

## Introduction

Data from the Centers for Disease Control and Prevention indicate that, in 2021, 38% of adults in the United States—amounting to approximately 98 million people—were living with prediabetes, a figure that increased to 49% among those aged 65 and older [1]. Globally, the International Diabetes Federation Atlas (10th Edition) estimated that 541 million adults had prediabetes in 2021, a number comparable to the global prevalence of diabetes. Prediabetes is a serious health concern, closely linked to metabolic syndrome and associated with increased cardiovascular risk and mortality [1, 2]. A 2020 meta-analysis of 129 studies including more than 10 million individuals found that, compared to normoglycemic individuals, those with prediabetes had an elevated relative risk of all-cause mortality, cardiovascular disease, coronary heart disease, and stroke over a median follow-up of nearly 10 years [3]. Since prediabetes represents the final stage before the onset of type 2 diabetes mellitus (T2DM), interventions that prevent or delay its progression offer a critical opportunity to curb the global diabetes epidemic.

The Physicians’ Health Study [4] previously reported a 14% reduction in the risk of incident diabetes over five years in a randomized trial of 325 mg aspirin every other day among apparently healthy men; however this finding did not achieve statistical significance. More recently, Zoungas and colleagues [5] demonstrated in a *post-hoc* analysis that daily low-dose aspirin modestly slowed the rise in fasting plasma glucose and reduced the incidence of T2DM by 15% among community-dwelling older adults, albeit with an increased risk of major bleeding. Notably, the difference in fasting glucose levels between the aspirin and placebo groups became apparent at all follow-up visits, and a separation in diabetes incidence curves emerged after the second year of treatment. These observations support the hypothesis that aspirin’s potential protective effect against incident diabetes may be particularly meaningful in individuals with prediabetes—a condition more common in the elderly and marked by elevated glucose levels. However, in the study by Zoungas et al. [5], diabetes diagnosis relied on self-reports, initiation of glucose-lowering therapy, or a single fasting plasma glucose value, without confirmatory testing or inclusion of HbA1c or oral glucose tolerance tests, thereby limiting the identification of prediabetes.

In light of aspirin’s potential role in lowering the risk of newly diagnosed T2DM in individuals with prediabetes, in the present cohort study we specifically assessed the impact of daily low-dose aspirin on incident T2DM in prediabetic adults living in Naples, Italy, who were followed by their primary care physicians between 2018 and 2022. These results were compared with a control group of individuals not treated with aspirin, derived from the same database. To account for baseline differences between the aspirin-treated and untreated groups, propensity score matching was employed based on age, gender, BMI, and smoking status.

## Methods

### Study design

We designed a longitudinal cohort study, utilizing data sourced from COMEGEN (“*COoperativa di MEdicinaGENerale*”: General Medicine Cooperative), a network of primary care physicians operating within the Naples Local Health Authority under the Italian Ministry of Health (“ASL Napoli 1 Centro”). Established in 1997, COMEGEN currently comprises 140 physicians, all interconnected through a shared electronic medical record system, forming an extensive repository of medical records for over 200,000 adult patients [6]. These records are continuously updated as each physician logs details of their outpatient activities daily.

The demographic composition of individuals managed by COMEGEN physicians closely reflects that of the general population as reported by the National Institute of Statistics (*ISTAT*), with no significant disparities in geographic or age distribution. Clinical diagnoses are recorded by primary care providers using ICD-10 codes, and all diagnostic procedures are documented using standardized codes. Pharmaceutical prescriptions are captured in detail, including the prescription date, brand name, active ingredients, dosage, and instructions for administration. The dataset also includes a wide range of clinical and health-related variables such as vital signs, anthropometric measures (including BMI and waist circumference), chronic conditions, healthcare utilization (consultations, hospitalizations, emergency visits), laboratory test results, medication dispensations, and mortality data, including date and cause of death. This comprehensive data infrastructure enables real-time tracking of patient care, clinical outcomes, medication use, diagnostic activity, and comorbidity burden. Accurate measurement of person-time is ensured through the recording of enrollment dates, death dates, follow-up completion, and observation endpoints for each patient.

Data were collected from January 1, 2018, to December 31, 2022, and demographic, clinical, laboratory, and pharmacological information was extracted from the COMEGEN database. This cohort study adhered to the STROBE reporting guidelines. Ethical approval was obtained from the Ethics Committee of “*ASL Napoli 1 Centro*”, which also granted a waiver of informed consent. The study was conducted under the patronage of the Italian Society for Cardiovascular Prevention (SIPREC). Exposure was defined as initiation of daily low-dose aspirin (100 mg), and the primary outcome was a new diagnosis of type 2 diabetes mellitus. At baseline, individuals with HbA1c > 6.5% (48 mmol/mol), a pre-existing diabetes diagnosis (ICD-10 codes E08.X–E13.X), or a history of antidiabetic medication use exceeding 30 days were excluded. Prediabetes was defined according to guidelines from American Diabetes Association (ADA) [7], WHO [8], and the International Expert Committee [9], based on fasting glucose levels of 100–125 mg/dL, a 2-h glucose value of 140–199 mg/dL following a 75 g oral glucose tolerance test, or HbA1c values between 5.7% and 6.4% or between 6.0% and 6.4%. Only individuals whose information about T2DM history was entirely available for 5-year follow-up and in which selected demographic and clinical data were available for the entire observation period were included in the study. For this reason, although individuals were able to join the cohort over time as they became eligible, they did so in only limited numbers.

*Inclusion criteria*: Age > 18-year-old; availability of information on diabetes history; availability of clinical and demographic data at least for 5 years.

*Exclusion criteria*: History of diabetes before 2018, cardiac ischemia, myocardial infarction, stroke, any site hemorrhage, heart failure, chronic kidney disease before 01.01.2018, other prescriptions of antiplatelet therapy, missing information on history, clinical, or demographic data.

In order to adjust for the difference in baseline characteristics between groups, clinically available predefined variables were used to define diabetic risk score. Predefined baseline variables included age, sex, body mass index (BMI), blood glucose measurements. Comorbidities such as cardiovascular disease, chronic kidney disease, hypertension, dyslipidemia were also included as predefined covariates since it has been reported that patients with cardiovascular disease, arterial hypertension or hyperlipidemia exhibit higher risks and burdens than people without these conditions [10].

### Outcomes

The main outcome of the study was the new diagnosis of T2DM assessed by the ICD-X codes E11 (“T2D”) and E13X (“Other specified diabetes mellitus”) defined as at least two measurements (not necessarily consecutive) of fasting plasma glucose concentration 126 mg/dl or higher or at least one HbA1c value of 6.5% or higher, or prescription of antidiabetic therapies for more than 30 days, as previously reported [11]. Diagnoses of T1D, including the specific code E08 were excluded.

Bleeding represented the safety outcome. We created a binary outcome based on an observation of at least one bleeding event, or no recorded bleeding event of the last available assessment. This outcome was designed to provide a conservative estimate of bleeding events during the follow-up. The involvement of the primary physicians allows also a reliable estimate of bleeding episodes of lesser severity which may have significant impact for patients but are difficult to capture. Bleeding complications were classified as severe or life-threatening if they were intracerebral or if they resulted in substantial hemodynamic compromise requiring treatment. Moderate bleeding was defined by the need for transfusion. Minor bleeding referred to other bleedings, not requiring transfusion or causing hemodynamic compromise, according to a previously reported bleeding classification [12].

### Statistical analysis

Data were summarized using mean and standard deviation for continuous variables and absolute frequency and percentage for categorical variables. Propensity score matching (PSM) was performed as we described [6, 13, 14] in order to balance 35,525 individuals by aspirin treatment groups (5066 under oral aspirin therapy and 30,459 without therapy, considered as control group). The PSM model included the following covariates: age, sex, BMI, statin therapy, antihypertensive therapy and prediabetes. Optimal matching was achieved using nearest neighbor matching with logit link and a caliper = 0.1, with a 1:1 ratio without replacement.

Mean differences were reported as Cohen’s D; risk difference was computed as difference between proportions for two independent populations. All differences are reported with 95% confidence intervals. Cox regression was used to identify association of aspirin with diabetes considered as time-to-event variable. The proportional hazard assumption was tested using the Schoenfeld residuals. The cumulative risk curves were constructed using the Kaplan–Meier method and comparison between curves is computed with log-rank test. Sensitivity analyses included the removal of all outliers and excluding covariates with more than 30% missing values. In order to confirm the results of the present analyses, sensitivity analyses were conducted. Firstly, all covariates with more than 30% missing values after outlier removal were not considered for the PSM model. Given the need of proceeding on complete cases for the application of PSM, and given the sparse presence of extreme values, we compared the results of three PSM models, i.e. on the dataset including outliers, removing cases with at least one outlier for a variable and replacing outlier values with a value imputed using multiple imputation. For all these analyses, after PSM, point estimates for Cohen’s D of the outcome variables were not considerably different, with groups including outliers having a slightly larger variability, as expected. Additionally, significant results were reproduced in all analyses. Thus, we proceeded with reporting results on complete cases, removing outliers and without imputation. Analyses were performed with R statistical software version 4.4.0. A p-value < 0.05 was considered significant for all analyses.

## Results

**Fig. 1:**
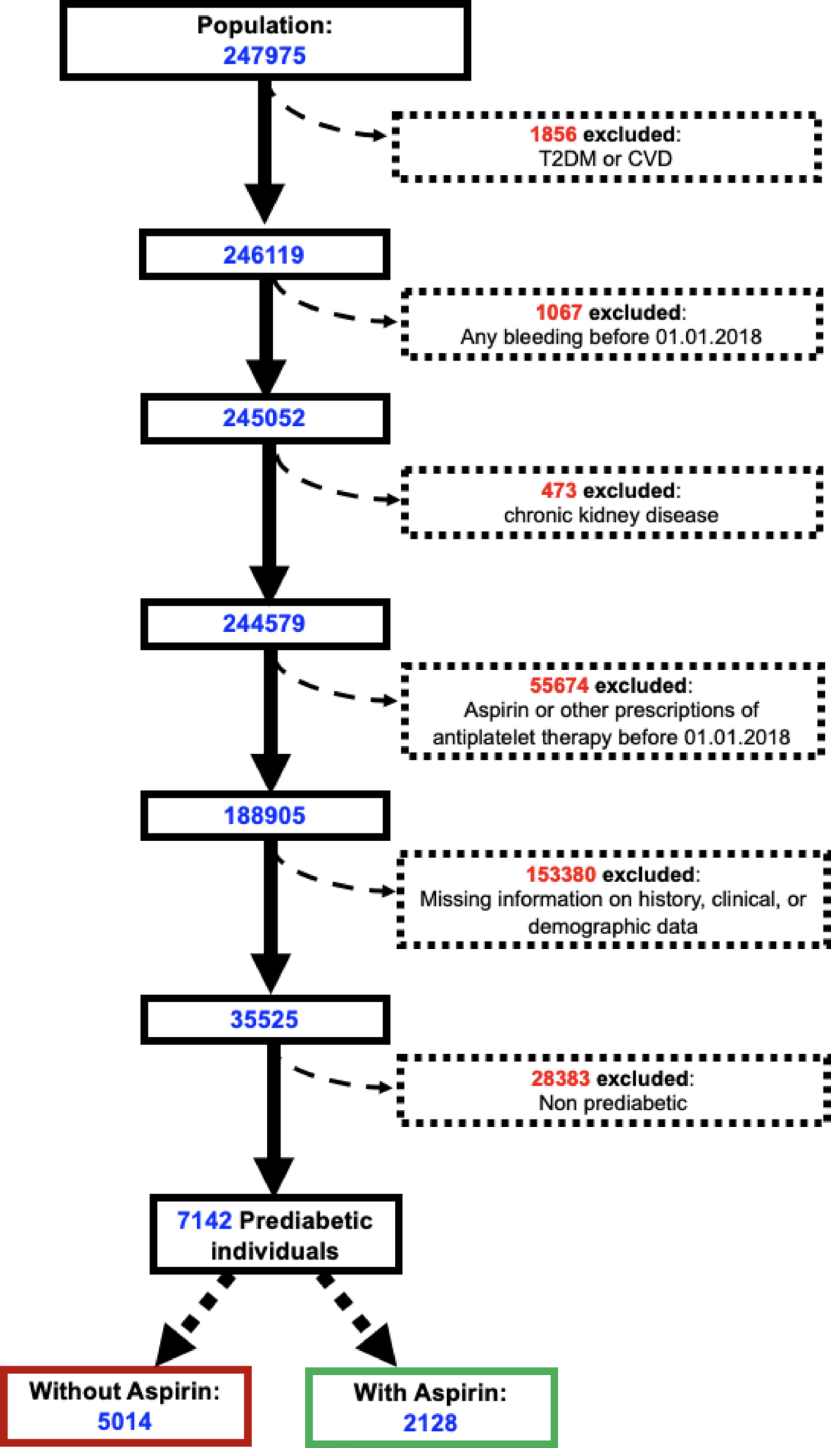
Flow chart showing the selection of the study population

Anonymized data from electronic records of 247,975 patients in the 2018–2022 period were considered for the study. After excluding individuals satisfying our exclusion criteria, we obtained anonymized data on a cohort of 35,525 participants between 2018 and 2022 (Fig. 1).

The aspirin treated population was significantly older and with significantly higher BMI; in addition, it had a significantly larger percentage of individuals with prediabetes or dyslipidemia or treated with statins or antihypertensive drugs (Table 1).

**Table 1:** Clinical and demographical characteristics of the two study groups according to aspirin treatment before matching

Variable	No-Aspirin N = 30,459	Aspirin N = 5,066	Difference	95% CI
Age (years)	57 (19)	78 (11)	− 1.2	**− 1.2, − 1.1**
Sex			0.07	**0.04, 0.10**
F	17,118 (56%)	2,677 (53%)		
M	13,341 (44%)	2,389 (47%)		
Dyslipidemia	11,266 (37%)	4,166 (82%)	− 45%	**− 46%, − 44%**
Prediabetes	5,014 (16%)	2,128 (42%)	− 26%	**− 27%, − 24%**
BMI (kg/m^2^)	25.8 (4.2)	27.1 (4.1)	− 0.32	**− 0.35, − 0.29**
Statins	7,841 (26%)	3,981 (79%)	− 53%	**− 54%, − 52%**
Antihypertensive therapy	8,604 (28%)	4,239 (84%)	− 55%	**− 57%, − 54%**

Bold values: significant difference (p<0.05)

Considering only prediabetes patients, the two groups with and without aspirin treatment were balanced for age, sex, BMI, treatment with statins and antihypertensive drugs using propensity score matching. The clinical and demographic characteristics of these groups after weighting are shown in Table 2. Evaluation of standardized mean differences and risk differences of these characteristics after weighting revealed no significant difference, suggesting good balance. At the final follow-up visit, concomitant use of medications, including anti-hypertensive drug classes, statins, other lipid-lowering drugs were comparable between the group treated with aspirin and the control group.

**Table 2:** Clinical and demographical characteristics of the two cohorts according to aspirin treatment after propensity score matching only in prediabetic patients

Variable	No-Aspirin N = 1,562	Aspirin N = 1,562	Difference	95% CI
Age (years)	75 (9)	76 (9)	− 0.07	− 0.14, 0.00
Sex			0.01%	− 0.06%, 0.08%
F	796 (51%)	803 (51%)		
M (%)	766 (49%)	759 (49%)		
BMI (Kg/m^2^)	27.5 (3.9)	27.6 (4.2)	− 0.01	− 0.08, 0.06
Statins (%)	1,207 (77%)	1,200 (77%)	0.45%	− 2.6%, 3.5%
Antihypertensives (%)	1,240 (79%)	1,241 (79%)	− 0.06%	− 3.0%, 2.8%

During the follow-up, 488 (15,6% of the study population) incident diabetes events were reported, of which 174 in the aspirin group (22.3 cases per 1000 person-years) and 314 in the control group (40.2 cases per 1000 person-years), thus the incidence of new T2DM was significantly lower in subjects with prediabetes treated with aspirin.

**Fig. 2:**
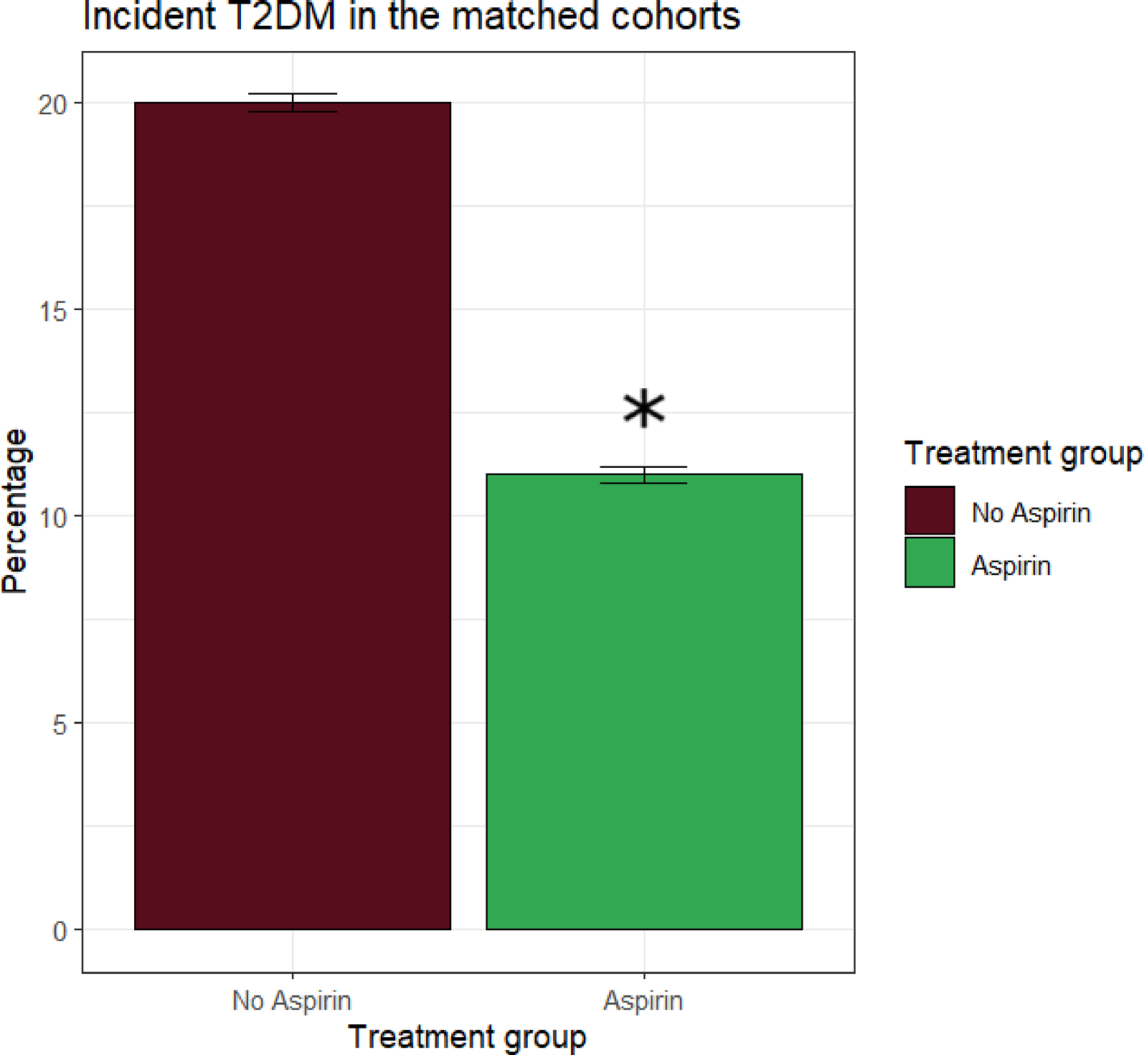
Graph showing the reduction of ~ 50% of the incidence of new T2DM in prediabetic patients treated with aspirin versu without aspirin after propensity score matching. Significant differences are denoted by asterisks (*)

The Cox model on the total follow-up period demonstrated a significant protective effect of aspirin compared with controls, since assignment to aspirin resulted in a 47% reduction in risk of incident T2DM (HR = 0.53 95% CI: 0.44–0.64, *p* < 0.001) (Fig. 2).

**Fig. 3:**
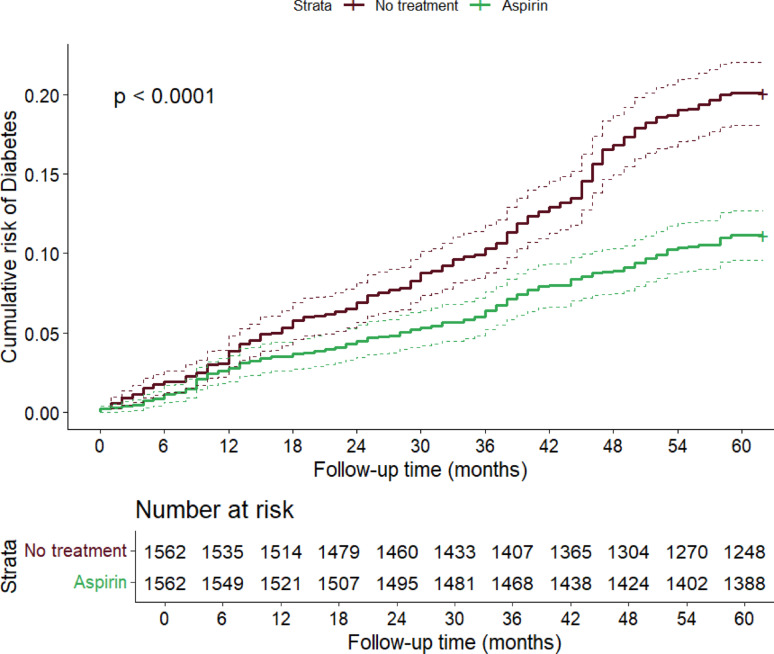
Difference in estimated cumulative risk of diabetes between aspirin treated and non-treated individuals. The curves were constructed with the Kaplan–Meier method and the *p*-value was computed via log-rank test. The table below shows COX regression model

Finally, Kaplan–Meier curves showed a significant difference in the cumulative risk of T2DM between the treated and not treated groups, with a more marked estimated risk difference in the numbers of T2DM cases after the second year of follow-up (Fig. 3), and an evident progressive increase in the difference between the curves (log-rank test *p* < 0.0001).

**Fig. 4:**
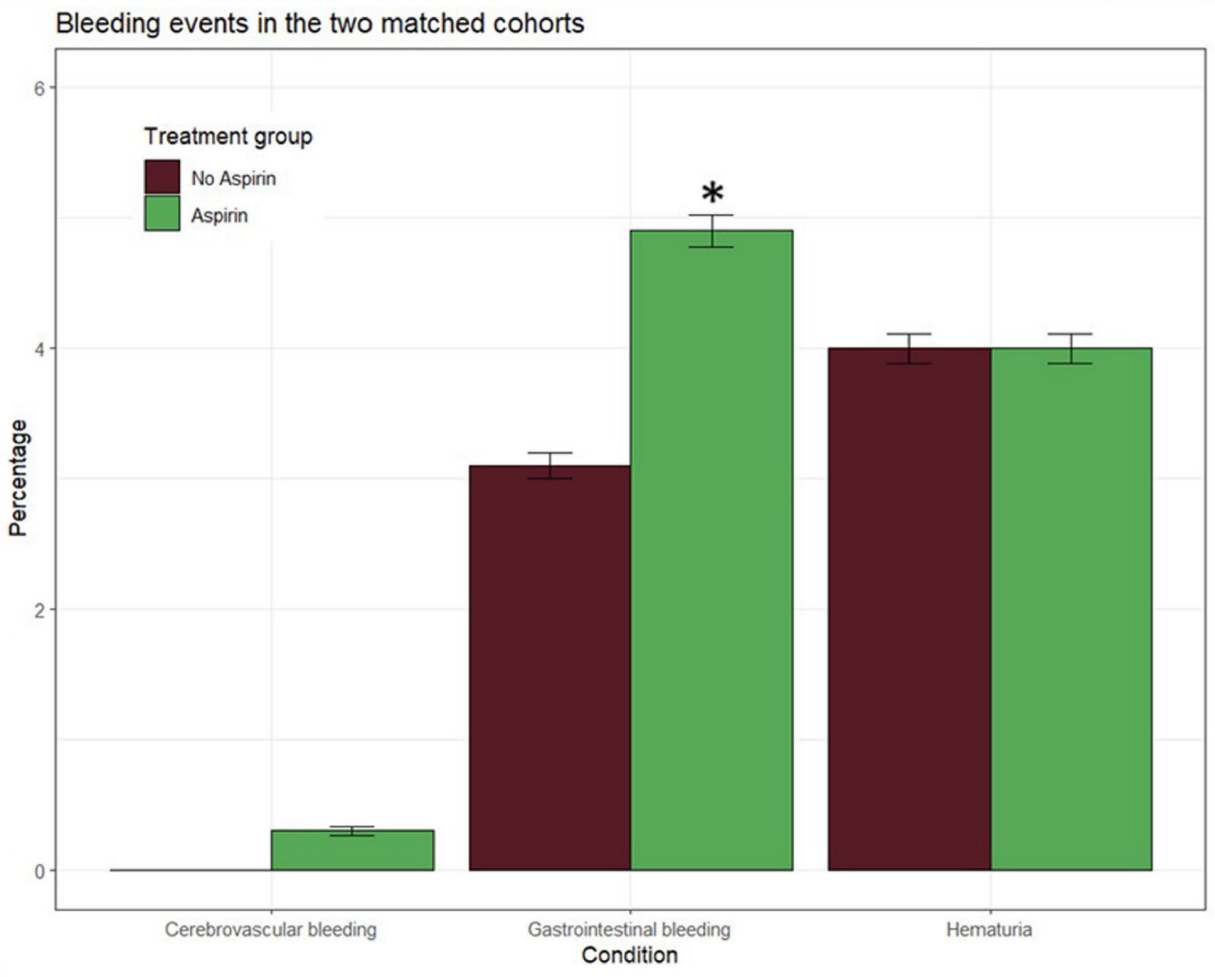
Graph showing bleeding incidence in prediabetic patients treated with aspirin versus without aspirin after propensity score matching. Significant differences are denoted by asterisks (*)

The safety analysis set included 3124 participants, with 1562 in the aspirin group and 1562 in the control group. Major bleeding occurred in 4 (0.2%) participants in the aspirin group and 3 (0.1%) participants in the control group. Moderate bleedings had an incidence rate of 9.8 events per 1000 person-years in the aspirin group and of 6.1 events per 1000 person-years in the control group. Finally, minor bleedings, represented by hematuria, were of 7.9 events per 1000 person-years in the aspirin group and 8.0 events per 1000 person-years in the control group. Compared with controls, aspirin treatment increased the risk of gastrointestinal bleedings (Table 3**, **Fig. 4).

**Table 3:** Observed events at follow-up in the two study groups according to aspirin treatment after propensity score matching only in prediabetic patients

Variable	No-Aspirin N = 1,562	Aspirin N = 1,562	Difference	95% CI
T2DM (%)	314 (20%)	174 (11%)	9.0%	6.4%, 12% *
Chronic CVD (%)	4 (0.3%)	7 (0.4%)	− 0.19%	− 0.67%, 0.29%
Myocardial infarction (%)	2 (0.1%)	14 (0.9%)	− 0.77%	− 1.3%, − 0.20% *
Heart failure (%)	16 (1.0%)	40 (2.6%)	− 1.5%	− 2.5%, − 0.54% *
Stroke (%)	4 (0.3%)	27 (1.7%)	− 1.5%	− 2.2%, − 0.72% *
Cerebrovascular bleeding (%)	1 (< 0.1%)	4 (0.3%)	− 0.19%	− 0.54%, 0.15%
Gastrointestinal bleeding (%)	48 (3.1%)	77 (4.9%)	− 1.9%	− 3.3%, − 0.42% *
Hematuria (%)	63 (4.0%)	62 (4.0%)	0.06%	− 1.4%, 1.5%

Significant differences are denoted by asterisks (*)

Bold values: significant difference (p<0.05)

## Discussion

The main finding of our study is the observation that treatment with a daily dose of 100 mg of aspirin reduces by ~ 50% the incidence of new diabetes but increases the risk of gastrointestinal bleeding in elderly subjects with prediabetes with a mean age of 78 years. To the best of our knowledge, our study is the first to be performed on a large sample of the general population and not on post-hoc analysis of a trial designed to address primary and secondary outcomes that did not include diabetes, or databases of hospitals or outpatient clinics. Indeed, in Italy all citizens have a primary care physician (family doctor) who follows them even if they do not have specific pathologies. Therefore, the database that we used includes not only patients with known illnesses but also individuals who do not have any disease (or at least are not aware of). With 5–10% of people per year with prediabetes progressing to T2DM, tackling the spiraling global epidemic of T2DM will only be possible with a renewed focus on and greater commitment to prevention programs targeting prediabetes and its progression, and our results suggest that it is possible to consider aspirin as a therapeutic strategy to face the diabetes pandemic.

The cumulative incidence curves suggest that the difference in T2DM onset between aspirin and non-aspirin users becomes more pronounced after the second year, a finding which is in agreement with the observation of Zoungas et al. (4) who reported that a divergence in case number beginning after the second year of aspirin or placebo treatment was shown in curves that plotted the cumulative incidence of T2DM. Given the proposed role of chronic subclinical inflammation in the development of insulin resistance [15] and the results of preclinical and clinical studies of salicylate treatment supporting an anti-inflammatory effect of both low and high doses [16–19], the time course of reduction of new T2D in our population of elderly subjects may allow to speculate that aspirin’s effect on inflammation plays a pivotal role in its genesis; future dedicate works might help to clarify the putative mechanisms of this phenomenon.

We are aware that the simultaneous increase in bleeding risk may represent a major limit to the use of aspirin for treatment of diabetes, however it is now well accepted that prediabetes is more than just a reading on a glycemic scale since it represents a condition in which cardiovascular risks became more pronounced when compared with normoglycemia especially in a population with pre-existing atherosclerotic cardiovascular disease (ASCVD) [1, 20–24]. Based on multiple clinical trials [25], the 2019 American College of Cardiology/American Heart Association guidelines on ASCVD primary prevention [26] narrowed recommendations on preventive aspirin use to those at higher ASCVD risk and 70 years old or younger without increased bleeding risk. Further attention to reducing low-value aspirin use in patients 60 years and older is warranted given 2022 recommendations by the US Preventive Services Task Force [27] which downgraded their recommendation on low-dose aspirin for primary CVD prevention based on an extensive systematic review concluding that the cumulated evidence for aspirin prevention against colorectal cancer was inconclusive and that primary prevention of CVD events closely matched bleeding harms [27–29]. However, a recent meta-analysis [30] showing that cardiovascular risks rose after stopping aspirin according to these recommendations has suggested that patients with a high risk of ASCVD might benefit from a daily low-dose aspirin when the advantages of preventing an initial cardiovascular event outweighed the harms associated with the chance of bleeding. Our findings may indicate that adults older than 70 years with prediabetes who are receiving low-dose aspirin to prevent ASCVD may also benefit from a slower progression to T2DM.

*Our study has several limitations*. First, we lacked data on over-the-counter (OTC) aspirin use, which may have resulted in the misclassification of individuals who used aspirin intermittently as non-continuous users. Second, the higher prevalence of cardiovascular events observed in the aspirin-treated group is unlikely to be attributable to the therapy itself, but rather may reflect residual confounding due to differing baseline cardiovascular risk profiles between groups, despite propensity score matching. Third, while our analysis focused on long-term consistent use of aspirin, we cannot rule out some degree of exposure misclassification due to potential nonadherence to prescribed therapy. Nevertheless, these limitations do not materially weaken the reliability of our findings. Finally, healthy user bias is a potential concern in observational studies evaluating long-term use of preventive medications. Individuals who adhere consistently to such treatments may also engage in other health-promoting behaviors that could influence outcomes. Albeit we were unable to directly assess health-seeking behavior, the greater burden of comorbidities and higher rates of concomitant medication use among low-dose aspirin users in our study argue against healthy user bias as a primary explanation for the observed associations.

In conclusion, our findings reinforce the association between long-term daily use of low-dose aspirin (100 mg) and a reduced incidence of newly diagnosed T2DM among individuals with prediabetes, although the precise antidiabetic mechanisms of aspirin remain to be elucidated.

## Data Availability

The datasets used and/or analysed during the current study are available from the corresponding author on reasonable request.
